# 100-nm-sized magnetic domain reversal by the magneto-electric effect in self-assembled BiFeO_3_/CoFe_2_O_4_ bilayer films

**DOI:** 10.1038/srep09348

**Published:** 2015-04-23

**Authors:** Keita Sone, Hiroshi Naganuma, Masaki Ito, Takamichi Miyazaki, Takashi Nakajima, Soichiro Okamura

**Affiliations:** 1 Department of Applied Physics, Faculty of Science, Tokyo University of Science, 6-1-3 Niijuku, Katsushika, Tokyo 125-8585, Japan; 2 Department of Applied Physics, Tohoku University, 6-6-05 Aoba, Aramaki, Aoba, Sendai 980-8579, Japan; 3 Department of Instrumental Analysis, Tohoku University, 6-6-11 Aoba, Aramaki, Aoba, Sendai 980-8579, Japan

## Abstract

A (001)-epitaxial-BiFeO_3_/CoFe_2_O_4_ bilayer was grown by self-assembly on SrTiO_3_ (100) substrates by just coating a mixture precursor solution. The thickness ratio of the bilayer could be controlled by adjusting the composition ratio. For example, a BiFeO_x_:CoFe_2_O_x_ = 4:1 (namely Bi_4_CoFe_6_O_x_) mixture solution could make a total thickness of 110nm divided into 85-nm-thick BiFeO_3_ and 25-nm-thick CoFe_2_O_4_. Self-assembly of the bilayer occurred because the perovskite BiFeO_3_ better matched the lattice constant (misfit approximately 1%) and crystal symmetry of the perovskite SrTiO_3_ than the spinel CoFe_2_O_4_ (misfit approximately 7%). The magnetic domains of the hard magnet CoFe_2_O_4_ were switched by the polarization change of BiFeO_3_ due to an applied vertical voltage, and the switched magnetic domain size was approximately 100nm in diameter. These results suggest that self-assembled BiFeO_3_/CoFe_2_O_4_ bilayers are interesting in voltage driven nonvolatile memory with a low manufacturing cost.

Non-volatile memories such as hard-disk drives (HDDs) and spin-transfer-torque magnetic random access memory (spin-MRAM) have been significantly developed over the last decade. The recording density of HDDs is the highest of the non-volatile memories used for commercial products. In general, the operation for writing these memories is carried out by applying an electrical current. From the viewpoint of electrical power consumption, the writing operation should essentially be performed by an electric field. Recently, the magnetic state of an ultrathin film was modulated by applying an electric field^1^, which can be used for the writing process by applying a voltage. Moreover, this modulation phenomenon is advantageous in terms of the device-fabrication process because it can use the process of magnetic tunnel junctions (MTJs)^2^. The manipulation of magnetic properties required a large voltage, and the thermal stability of the magnetic layer might be small because the layer is ultrathin. Therefore, these two issues need to be resolved before practical application becomes possible. Another approach is to utilize multiferroic materials as one way to generate the voltage required for the writing process. Spin-frustration type multiferroics such as *R*MnO_3_ (where *R* = Tb, Dy, *etc*.) and CuFeO_2_ have ferroelectric and magnetic-order parameters^3^,^4^,^5^. These multiferroics exhibit breaking of space inversion symmetry by magnetic ordering, and this broken symmetry induces ferroelectricity, which implies strong magneto-electric (ME) coupling. However, most multiferroics show transition temperatures lower than room temperature (RT). BiFeO_3_ is a one of the very few multiferroic materials that has a ferroelectric Curie temperature of 850C and an antiferromagnetic Nel temperature of 370C. The ME effect between antiferromagnetism and spontaneous polarization owing to the Dzyaloshinskii-Moriya (DM) interaction by an asymmetric crystal structure has also been observed at RT. In the case of a BiFeO_3_/ferromagnetic bilayer, magnetization switching was clearly observed at RT through an antiferromagnetic coupling^6^ by reversal of polarization^7^. In this report, an electric field was applied to BiFeO_3_ in the horizontal direction; therefore, a large voltage had to be applied to reverse the polarization. A voltage applied in the vertical direction can be reduced compared with one applied in the horizontal direction. When BiFeO_3_ is used as a tunnel barrier in MTJs, the writing operation performed by an electric field is carried out in the vertical direction. However, magnetization switching by polarization reversal using a bilayer structure under applied vertical voltage has been investigated in a few works^8^.

As for the process for fabricating solid-state memory devices, most are fabricated using an ultra-high-vacuum process; therefore, expensive fabrication equipment is necessary. If these devices could be fabricated using a wet chemical process, the low cost of investment in facilities at the start of research would be an advantage. In addition, using spray coating or chemical solution deposition (CSD) would lower the fabrication cost per unit. In a previous report, when a Bi-rich BiFeO_3_ target was used, BiFeO_3_ was epitaxially grown on the SrTiO_3_ (001) substrate, and excess Bi was grown on the BiFeO_3_ as Bi_2_O_3_^9^. Due to the large lattice mismatch between Bi_2_O_3_ and SrTiO_3_ compared with that of BiFeO_3_ and SrTiO_3_, Bi_2_O_3_ formed at the surface of the film. This result suggested that by adjusting the lattice misfit between a SrTiO_3_ substrate and a ferromagnet or BiFeO_3_, a BiFeO_3_/ferromagnet bilayer can be prepared even by a one-time-only liquid phase process. CoFe_2_O_4_ is a candidate material for a ferromagnetic layer because the lattice mismatch between CoFe_2_O_4_ and SrTiO_3_ is 7.3%, which is much larger than that between BiFeO_3_ and SrTiO_3_ (1.4%). These material combinations are expected to enable fabrication of a BiFeO_3_/CoFe_2_O_4_ bilayer on a SrTiO_3_ substrate by utilizing the differences in lattice mismatch. Whether the magnetization can be reversed by the ME effect of BiFeO_3_ when using materials with high-magnetic-anisotropy has yet to be verified in the case of a bilayer system. Without that verification, it is not possible to apply BiFeO_3_ to high-density memories. From the viewpoint of the magnetocrystalline anisotropy energy (*K*_u_), CoFe_2_O_4_ is one of the candidate hard magnetic materials (*K*_1_ of approximately 3 10^6^erg/cm^3^ in bulk). Self-assembled CoFe_2_O_4_-BiFeO_3_ (or BaTiO_3_) epitaxially grown on SrTiO_3_ substrates using pulsed laser deposition (PLD) has been reported^10^,^11^,^12^,^13^,^14^,^15^,^16^. In many of these reports, the CoFe_2_O_4_ nanopillars were embedded in the BiFeO_3_ matrix; namely, it is a nano-composite structured film. The CoFe_2_O_4_ nanopillars were complexly influenced by the exchange bias from the BiFeO_3_ matrix; moreover, they might be influenced by a strain effect^13^ from the BiFeO_3_ because a nanopillar has a degree of freedom to move in the vertical direction of the film. In the case of a bilayer structure, the ME effect becomes simpler than that in the case of a nanopillar structure. However, polarization reversal using a BiFeO_3_/CoFe_2_O_4_ bilayer has not been phenomenologically investigated much in terms of magnetization switching. In the meantime, the minimum magnetic domain size in the case of a layered structure is not clear. It can be considered that the growth of the PLD process is not strongly influenced by the differences between the lattice misfits of CoFe_2_O_4_ and SrTiO_3_ substrates because, for example, PLD can prepare a non-equilibrium phase. A wet chemical process of thermal equilibration (such as CSD) is expected to more effectively expose the influence of lattice misfit of materials.

In this study, we demonstrated a novel chemical solution method; namely, introducing lattice misfit in relation to a single crystal SrTiO_3_ substrate using a BiFeO_3_/CoFe_2_O_4_ bilayer film, is proposed. The BiFeO_3_/CoFe_2_O_4_ bilayer was microfabricated as a vertical device structure to reduce the operation voltage based on the ME effect, and it was experimentally verified that CoFe_2_O_4_ magnetic domains (of 100-nm-diameter scale) with relatively high magnetocrystalline anisotropy could be switched.

Enhanced metalorganic decomposition (EMOD) solutions (Kojundo Chemical Laboratory Co., Ltd.) were used in this study. Two metal-diethylhexanoate compositions were used as the starting precursor solutions. As for the first, the ratio of Bi^3+^ and Fe^3+^ precursors was even in terms of atomic percent (*P*_BFO_) (i.e., a typical condition for preparing BiFeO_3_ films). For the second, the ratio of Co^2+^ and Fe^3+^ precursors was 1:2 in terms of atomic percent (*P*_CFO_) (i.e., a typical condition for preparing CoFe_2_O_4_ films). Each precursor solution was mixed to form an atomic percent ratio (*P*_BFO_:*P*_CFO_) of 4:1. The mixed precursor solution ratio (%), i.e., Bi^3+^:Fe^3+^:Co^2+^, was 36:55:9. The solution was spin-coated at 6000rpm for 50sec on a 5-at.% La-doped SrTiO_3_ (La-SrTiO_3_) (001) conductive single crystal substrate. The spin-coated films were dried at 150C for 1min and calcined at 350C for 5min using hot plates in air. This process was repeated six times. Sintering for crystallization was carried out at 650C for 10min in air using an infrared lamp heating system. Circular Pt top electrodes (with a diameter of 100m and a thickness of 60nm) were deposited on the film surface by DC magnetron sputtering. The crystal structures of the films were evaluated by X-ray diffraction (XRD; Philips, Xpert-Pro MRD). The cross sections of the films were observed by transmission electron microscopy (TEM; Hitachi HF-2000, FEI Company Tecnai G^2^ F20). The ferroelectric properties of the films were evaluated at 90K using a ferroelectric tester (TOYO Corporation, FCE-1A). The temperatures of the films were measured using a thermocouple contacted with silver paste. The ferroelectric domains were observed by piezoelectric force microscopy (PFM; Asylum Technology Cypher). Various poling methods were carried out before ferroelectric measurement. The magnetic hysteresis loops were measured using a superconducting quantum interference device (SQUID; Quantum Design MPMS). The magnetic domains were observed by magnetic force-microscopy (MFM; Bruker AXS Digital instruments, NanoScope IVa, Dimension 3100 stage AFM system).

The cross-sectional TEM observations of the film on the La-SrTiO_3_ (100) substrate are shown in Figs. 1(a) to 1(g). A bright-field TEM image is shown in Fig. 1(a). A slight bumpy contrast between the film surface (bright) and film body (dark) was obvious. The nanobeam electron diffraction patterns of the regions indicated by circles (i) to (iv) in Fig. 1(a) are shown in Figs. 1(b)1(e), respectively. At the surface area, randomly oriented CoFe_2_O_4_ diffraction spots were observed in Fig. 1(b). BiFeO_3_ diffraction spots were observed in Fig. 1(d) and these diffraction spots corresponded to those of the La-SrTiO_3_ (100) substrates Fig. 1(e). This correspondence indicated that BiFeO_3_ was epitaxially grown on the La-SrTiO_3_ substrates in a cube-on-cube crystal relationship (under the assumption of a pseudo-cubic-perovskite structure in BiFeO_3_). Both epitaxial BiFeO_3_ and randomly oriented CoFe_2_O_4_ diffraction spots coexisted at the interface Fig. 1(c). The high-resolution TEM image in Fig. 1(f) revealed that the interface between CoFe_2_O_4_ and BiFeO_3_ was clear and no intermixing interfacial layer of these two materials occurred. It should be noted that although a mixture precursor solution was used and the film was deposited only once, the film was separated into two layers, namely, an epitaxial BiFeO_3_ layer and a polycrystalline CoFe_2_O_4_ layer. The lattice misfit between BiFeO_3_ and the SrTiO_3_ substrate (1.4%) is smaller than that between CoFe_2_O_4_ and the SrTiO_3_ substrate (7.3%). It is therefore considered that the small lattice misfit of BiFeO_3_ was the reason for its precedential growth in regard to CoFe_2_O_4_, which meant that bilayer samples can be produced by utilizing the lattice mismatch with respect to the substrate materials. We believe that bilayer films formed instead of nanopillar composite films^9^,^10^,^14^ because crystal growth by CSD is in a thermal equilibrium state in comparison with that by PLD; as a result, the influence of the differences between lattice misfits of the substrates and epitaxial films was strong in the case of CSD. The total film thickness was estimated to be 110nm, and the thicknesses of the BiFeO_3_ and CoFe_2_O_4_ layers were estimated to be 85nm (*t*_BFO_) and 25nm (*t*_CFO_), respectively. It is noteworthy that the thickness ratio (*t*_BFO_:*t*_CFO_ = 3.4:1.0) approximately corresponded to the precursor-solution ratio under the assumption that the ratio of BiFeO_3_ and CoFe_2_O_4_ was given as *P*_BFO_:*P*_CFO_ = 4.0:1.0. This result indicated that the thickness ratio of BiFeO_3_ and CoFe_2_O_4_ can be controlled by adjusting the composition ratio of *P*_BFO_ and *P*_CFO_. The ** - 2** X-ray diffraction (XRD) pattern is shown in Fig. 1(h), and grazing incident XRD (GI-XRD) patterns are shown in Figs. 1(i) and 1(j). The incident angle was fixed at 1.5 for the GI-XRD measurements. The diffractions by the (311) and (440) planes related to CoFe_2_O_4_ and indicated random orientation of the CoFe_2_O_4_ layer, which was consistent with the TEM analyses. Figs. 1(b) and 1(c) The positions of the (311) and (440) diffraction patterns corresponded to the bulk CoFe_2_O_4_ diffraction angles, indicating that CoFe_2_O_4_ in the BiFeO_3_/CoFe_2_O_4_ bilayer film was not strained in the as-grown state. BiFeO_3_ showed an ME effect based on the switching of three different polarizations due to its rhombohedral distorted BiFeO_3_^7^,^8^. In the case of tetragonal symmetry, only polarization of the (001) plane switched, and symmetry of the antiferromagnetic plane does not change; therefore, the ME effect does not occur. The crystal symmetry of BiFeO_3_ is strongly influenced by the type of substrate, and the conditions of the sputtering; therefore, determine the crystal symmetry in film form is a key factor in using the ME effect. X-ray reciprocal space maps (RSMs) around the 004 and 204 spots are shown in Figs. 1(k) and 1(l), respectively. When BiFeO_3_ has a tetragonal symmetry, only one spot is observed in the 204 RSM regions,and two spots are observed along the *Q_z_* axis if BiFeO_3_ has a rhombohedral symmetry. The split of diffraction spots related to the rhombohedral symmetry were clearly observed; thus, the BiFeO_3_ layer had a rhombohedral crystal symmetry with a space group of *R*3c. The lattice parameters of BiFeO_3_ (estimated from the RSMs) were *a* = 0.396nm and ** = 89.5, which corresponded to those of bulk BiFeO_3_^17^.

The polarization-electric field (*P*-*E*) hysteresis loops of the (001)-epitaxial-BiFeO_3_/CoFe_2_O_4_ bilayer film is shown in Figs. 2(a). The*P*-*E* loop with a rounded shape measured at 2kHz became sharp as frequency increased above 5kHz. The leakage current was linear as a function of time; however, ferroelectric switching occurred within a few tens of nanoseconds. Sharp *P*-*E* loops were therefore obtained at high frequency due to the reduction of the leakage current. The polarization value of the BiFeO_3_/CoFe_2_O_4_ bilayer film estimated from the 20kHz loop was 91C/cm^2^. Toconfirm the polarization of the BiFeO_3_/CoFe_2_O_4_ bilayer film, the electrical displacement was measured by the positive-up-negative-down (PUND) method. Fig. 2(c) Schematic illustrations of the PUND responses for positive and up pulses are divided into four components: spontaneous polarization (SPC), initial increment (IC), paraelectric (PC), and leakage (LC) components. Fig. 2(d) For comparison of time scale, the wave forms of the applied voltage to the samples for the *P*-*E* (2kHz) and PUND are illustrated in Fig. 2(b). SPC and PC increased when the electric field applied, and LC linearly increased. SPC remains; however, PC and LC disappear when the electric field was removed. SPC can be calculated by subtracting PC from IC. It is considered that the PUND method may express spontaneous polarization more accurately than with the *P*-*E* loops measured by a ferroelectric tester. The PUND measurement was discussed in detail elsewhere^18^. The spontaneous polarization evaluated by PUND was 84C/cm^2^, which is almost same as that evaluated by *P*-*E* loops. The spontaneous polarizations determined by the different ferroelectric measurement methods coincided; thus, it can be considered that the polarization of BiFeO_3_ in the BiFeO_3_/CoFe_2_O_4_ bilayer film was completely switched by the applied electric field. The magnetization *v.s.* magnetic field (*M*-*H*) curves of the BiFeO_3_ and BiFeO_3_/CoFe_2_O_4_ bilayer films are shown in Fig. 2(e). The inset shows the entire hysteresis loop. The saturated magnetization (*M*_s_) and magnetic coercive field (*H*_c_) were estimated to be 118emu/cm^3^ and 510Oe, respectively, which were consistent with previously reported values^19^ for polycrystalline BiFeO_3_-CoFe_2_O_4_ nano-composite films. The *P*-*E* and *M*-*H* hysteresis measurements showed that the electrical and magnetic properties of the BiFeO_3_/CoFe_2_O_4_ bilayer film originated in BiFeO_3_ and CoFe_2_O_4_, respectively.

The ferroelectric switching characteristics of the local area and the influence of the surface potential on the magnetic stray field were evaluated by scanning probe microscopy (SPM). A PFM phase loop and amplitude loops taken by switching spectroscopy (SS) are shown in Fig. 3(a) and Fig. 3(b) for the BiFeO_3_/CoFe_2_O_4_ bilayer film. A SS-PFM phase loop and amplitude loop are shown in Fig. 3(e) and Fig. 3(f), and the PFM phase mapping image and amplitude mapping image are shown in Fig. 3(g) and Fig. 3(h) for a 90-nm-thick (001)-epitaxial-BiFeO_3_ film. The PFM information corresponded to the vertical response, and the SS-PFM loops were the result of the second loop of two continuous measurements. The 90-nm-thick (001)-epitaxial-BiFeO_3_ film had ferroelectric domains varying in diameter between several dozen and one hundred nanometers. For example, a relatively small ferroelectric domain (diameter of 60nm) with respect to the vertical direction was measured by SS-PFM. Figs. 3(e) and 3(f) The switching electric fields estimated by the SS-PFM phase and amplitude loops were the same, namely, -0.6 and 0.4MV/cm, respectively, which produced negative shift of approximately 0.1MV/cm. The shift of the SS-PFM loops might be attributed to the charge injection at the one-side interface^20^ due to the low measurement frequency (0.3Hz) of the SS-PFM method. Fig. 3(i)^21^ The SS-PFM phase loops showed that the ferroelectric domain showed a small coercive electric field compared with that observed in the *P*-*E* hysteresis loops. In the case of the *P*-*E* measurement, the diameter of the Pt electrode was 100m which included the various types of ferroelectric domains. In contrast, the SS-PFM measurement selected the ferroelectric domains having active responses to the vertical direction, which might be the reason for small coercive electric field in SS-PFM to the vertical direction compared with those obtained from the *P*-*E* measurement. Before the ME effect was measured, images of the BiFeO_3_/CoFe_2_O_4_ bilayer film were taken using MFM and Kelvin force microscopy (KFM). Figs. 3(e) and 3(f) MFM is detected in the tapping mode of the phase shift of the resonance frequency of the cantilever due to attractive (or repulsive) forces between the magnetized cantilever and the magnetic moment at the film surface. AFM and KFM are also used in tapping mode to observe the surface morphology and surface electrical potential, respectively. To clarify the influence of the surface electrostatic potential and the surface morphology on the MFM measurement, first obtained the surface morphology image by AFM, and then the cantilever distant from surface, KFM measurements were performed using an electrical feedback circuit. Finally, MFM measurements were carried out. The difference in the contrasts of the MFM image and the KFM image revealed that the surface electrostatic potential was not necessary taken into consideration for the ME effect measurement.

To reduce the switching voltage and understand the phenomenology of the ME effect in the vertical direction, a two-step electrode was designed. A thick top electrode is necessary to prevent penetration of an electrode by the detection needle used for measuring the ferroelectric polarization reversal, where a thin electrode is necessary for MFM to detect the stray-magnetic-field signal. To solve these two contradictory matters, the needles for detecting the ferroelectric polarization reversal were connected to 60-nm-thick electrodes, and the MFM tips were connected to 5-nm-thick electrodes. A two-step Pt electrode was prepared by manually shifting the shadow mask slightly when the Pt electrode was sputtered for the first and second times. A schematic diagram of the setup for evaluating the ferroelectric and magnetic properties is shown in Fig. 4(a). The reversal of the ferroelectric domains in the 60-nm-thick electrode area was confirmed by investigating the relationship between the electrode size and the polarization reversal charge. For theME measurements, the polarization of BiFeO_3_ was switched in the upward direction by applying a voltage of 20V (1.8MV/cm), and then the magnetization reversal was observed by MFM. As described in Fig. 2(a), 20V (1.8MV/cm) could reverse the ferroelectric polarization. To confirm the polarization reversibility, the polarization of BiFeO_3_ was switched in the downward direction, and the magnetization reversal in the same area was again observed by MFM. The switched polarization in BiFeO_3_ was stable for several weeks at RT in air. MFM observation was carried out in taping mode with a CoCr coated cantilever, and the magnetization direction (magnetic north) of the cantilever was upward to the film plane. The space resolution deduced from the digital data points (256 points/1.0m) is 4nm. In fact, the space resolution of MFM observation depends on the distance between the cantilever and the film surface; therefore, the actual space resolution was a few tens of nanometers. In order to reduce the influence of the atomic force in tapping mode, the distance of the cantilever was slightly increased during MFM observation compared with AFM observation. The gap between the cantilever and the film surface was kept at approximately 20nm during the MFM observation; that is, the actual distance between the CoFe_2_O_4_ and the cantilever was approximately 25nm. The observed area was 2.0 by 2.0m. MFM images of CoFe_2_O_4_ with upward and downward polarizations of BiFeO_3_ are shown in Figs. 4(b) and 4(c), respectively, and the subtracted upward- and downward-MFM images are shown in Fig. 4(d), which showed many dot-like contrast changes. Here, the influence of piezoelectric strain due to the polarization reversal in BiFeO_3_ domains on magnetization in CoFe_2_O_4_ was explained as follows. Piezoelectric intrinsically cannot conserve their strain without an electric field; therefore, a large piezoelectric constant is necessary for the polarization reversal. The piezoelectric constant (*d*_33_) estimated from the slope of the SS-PFM amplitude in the case of the BiFeO_3_ and CoFe_2_O_4_/BiFeO_3_ bilayer films was approximately 15pm/V and 10pm/V, respectively were smaller than the reported range of 50 to 100pm/V^22^. The piezoelectric strain under an applied electric field of 1.0MV/cm was less than 1%, which meant that the piezoelectric strain from the BiFeO_3_ layer could not reverse the magnetization of CoFe_2_O_4_. It may be noted that interface of the CoFe_2_O_4_ and BiFeO_3_ layers was slightly wavy, Fig. 1(a) which might make the strain influence on the ME effect enhanced at the interface edge. It can thus be considered that the magnetization switching of CoFe_2_O_4_ was basically derived from polarization switching of BiFeO_3_ and exchange coupling between antiferromagnetic BiFeO_3_ and ferromagnetic CoFe_2_O_4_; moreover, the strain effect at the edge of interfaces possibility enhanced theME effect. In Fig. 4(d), the polarization was partially reversed; however, the contrast did not change across the whole area because the BiFeO_3_ epitaxial film has three ferroelectric domains and only the 180 ferroelectric domains can not switch the magnetization by the ME effect. Therefore, the 71 and 109 ferroelectric domains might have respond to the ME effect^23^,^24^. Another possible reason is that even though the magnetization was switched by the 71 and 109 domains, the magnetization change of CoFe_2_O_4_ was not always in a state that was not directly detected as a change in the cantilever direction of MFM; Enlarged MFM images with typical upward and downward polarizations area, indicated as green squares (i) in Figs. 4(b) and 4(c), are shown in Figs. 4(e) and 4(f). The gradation of brown contrast differed in the cases of upward and downward polarization. Line profiles taken from the areas of the MFM images indicated as green squares (i), (ii), and (iii) are shown in Fig. 4(g). These line profiles showed that the magnetic domains were reversed by the electric field in three areas; in particular, the smaller magnetic domains indicated by areas (i) and (ii) were approximately 100nm in diameter. As mentioned above, the actual space resolution of MFM was a few tens of nanometers; therefore, a magnetization-switching signal from an area with diameter of approximately 100nm was within the measurement range. As for the size of a magnetic domain Fig. 4(d), the minimum size of a switched magnetic domain was around a few dozen nano meters in diameter. These reversed magnetic domain sizes were roughly consistent with ferroelectric domain size of BiFeO_3_. Figs. 3(a) and 3(c) The ferromagnetic and ferroelectric domains seemed to couple in a one-to-one relationship^8^. However, the MFM resolution is lower than that of the PFM contact measurement; accordingly, small magnetic domains are necessary for further investigation. In this study, the relatively large magnetocrystalline anisotropy of CoFe_2_O_4_ could be reversed by applying an electric field through exchange bias, and this result indicated that materials with higher magnetocrystallinity (such as an *L*1_0_-ordered alloy) can be expected to be applied to solid-state memories in the near future.

BiFeO_3_/CoFe_2_O_4_ bilayer films were formed on La-SrTiO_3_ (001) substrates by a one-time-only liquid phase process that involved spin coating of a mixed precursor solution. Cross-sectional TEM analysis confirmed that the BiFeO_3_ was epitaxially grown on the La-SrTiO_3_ substrates and that polycrystalline CoFe_2_O_4_ was grown on the BiFeO_3_ layer. The bilayer could be formed by an all-at-once chemical process in which BiFeO_3_ (*i.e.*, not CoFe_2_O_4_) preferentially grew on the La-SrTiO_3_ (001) substrate because the lattice mismatch between BiFeO_3_ and La-SrTiO_3_ is much smaller than that between CoFe_2_O_4_ and La-SrTiO_3_. Two-step top electrodes were used to evaluat the ME effect generated by applying a vertical electric field. The orientation of the small magnetic domains of CoFe_2_O_4_ changed when the polarization of BiFeO_3_ was switched to the opposite direction by applying a voltage. The key points regarding the ME effect are twofold: a material such as CoFe_2_O_4_ with relatively large magnetocrystalline anisotropy could be switched through ME coupling with BiFeO_3_, and the orientation of the magnetic domains of CoFe_2_O_4_ (namely, 100nm in diameter) could be reversed. These results suggest that a novel, low power, high-density MRAM and HDDs, which can be written by applying a voltage, can be created on the basis of the two points described above.

## Figures and Tables

**Figure 1:**
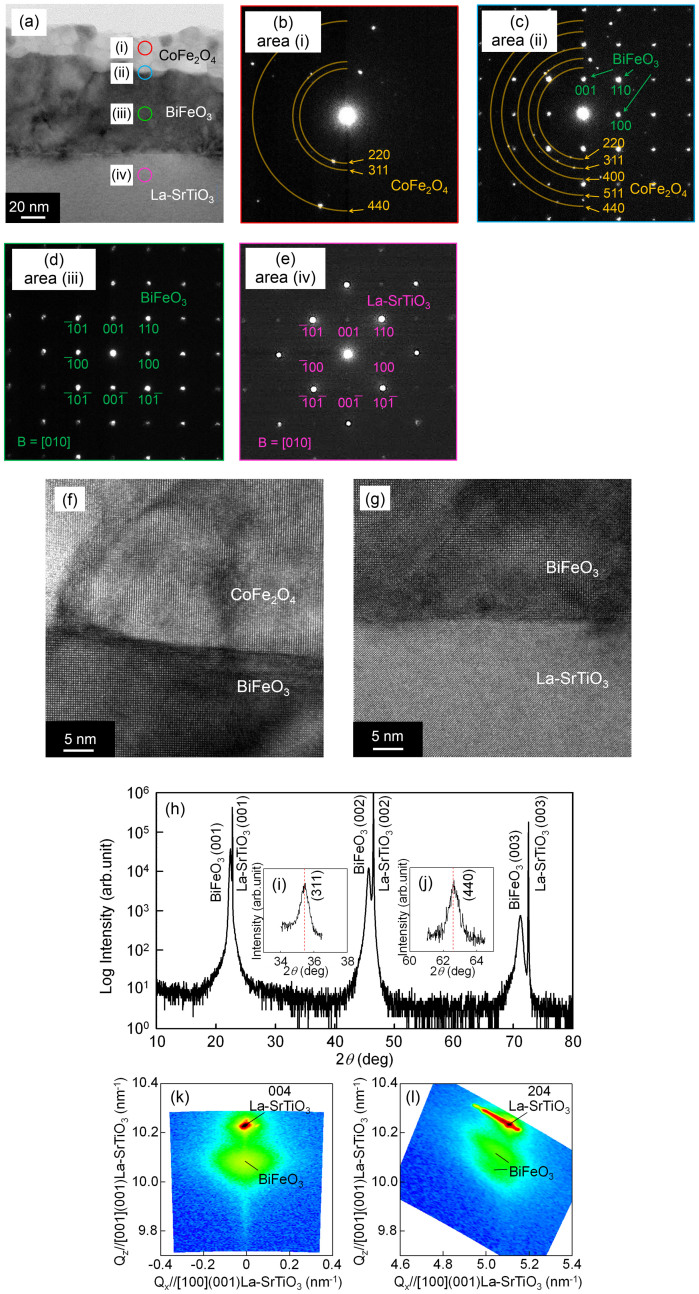
(a) Cross-sectional TEM image of a wide area of the sample, (b)(e) electron-diffraction patterns taken from circles (i)(iv), and cross-sectional high-resolution TEM images taken at the (f) BiFeO_3_/La-SrTiO_3_ and (g) CoFe_2_O_4_/BiFeO_3_ interfaces. (h) Out-of-plane /2 XRD pattern, GI-XRD patterns around (i) (311) and (j) (440) of the CoFe_2_O_4_ diffraction peaks, and RSMs around the (d) 004 and (e) 204 spots of the (001)-epitaxial-BiFeO_3_/CoFe_2_O_4_ film.

**Figure 2:**
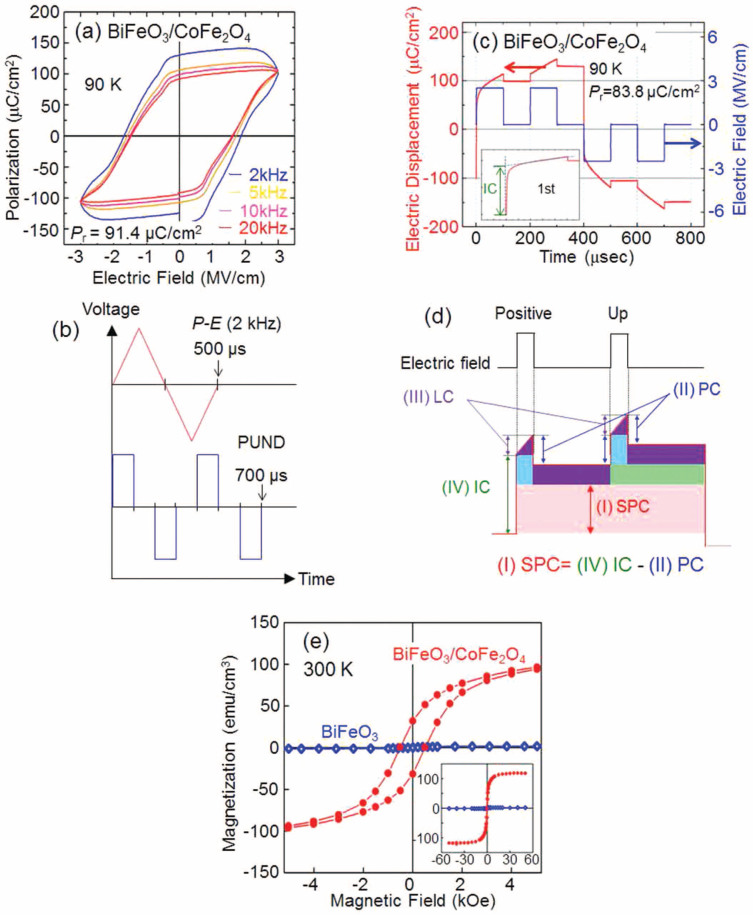
(a) *P*-*E* hysteresis loops of the (001)-epitaxial-BiFeO_3_/CoFe_2_O_4_ bilayer film at 90K at various measurement frequencies, (b) illustration of wave-forms when applying voltage to the samples for ferroelectric measurement, (c) positive-up-negative-down (PUND) response of the BiFeO_3_/CoFe_2_O_4_ bilayer film at 90K and (d) schematic illustration of the PUND response for ferroelectric materials with coexisting paraelectric and leakage components. SPC = IC PC^16^. (e) *M-H* hysteresis loops of the (001) epitaxial BiFeO_3_ and the BiFeO_3_/CoFe_2_O_4_ bilayer film measured at 300K. The inset shows the entire hysteresis loop.

**Figure 3:**
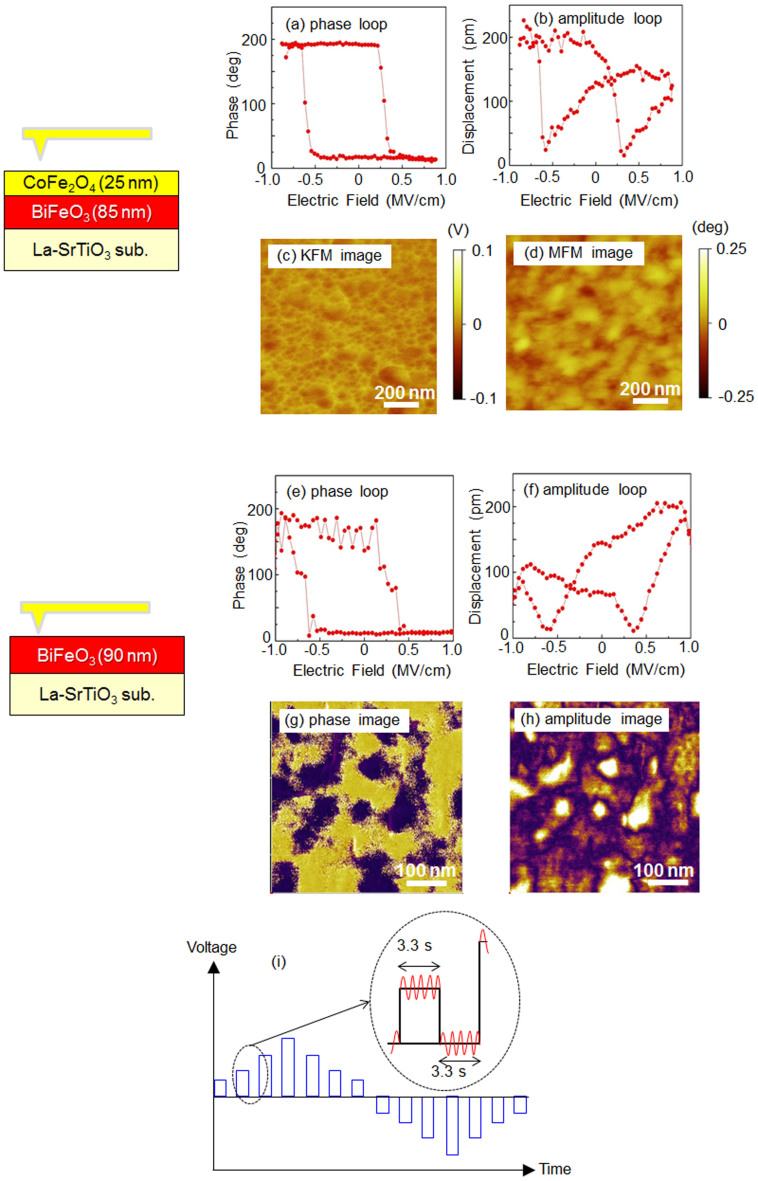
(a) and (b) SS-PFM phase and amplitude loops of the BiFeO_3_ (85nm)/CoFe_2_O_4_ (25nm) bilayer film. The surface potential and stray magnetic field were measured by (c) KFM and (d) MFM of the BiFeO_3_/CoFe_2_O_4_ bilayer film. MFM and KFM images of the same area were taken, and the contrasts in the magnetic and surface electrostatic potentials were different. The SS-PFM (e) phase loop and (f) amplitude loop, and their PFM mapping images (g) and (h) of a 90-nm-thick BiFeO_3_ epitaxial film. (i) Illustration of the voltage wave form v.s. time.

**Figure 4:**
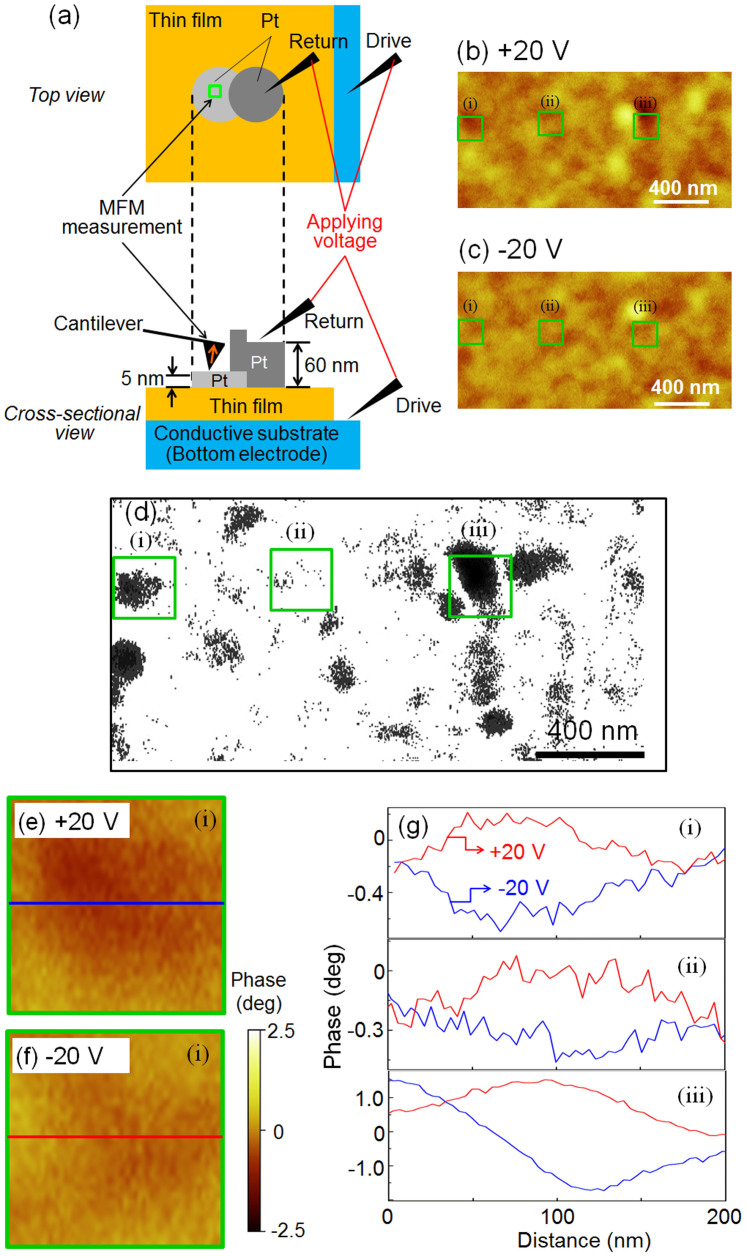
(a) Schematic diagram of evaluation of the ME effect; MFM images of a domain with CoFe_2_O_4_ for (b) upward and (c) downward polarizations. (d) The black-contrast area indicates a stray field changed by a polarization reversal. (e) and (f) Enlarged MFM images of the area marked by green squares (i) indicated in Figs. 4(b) and 4(c). (g) MFM line profiles taken from the upward and downward polarized areas (i), (ii), and (iii).
